# Cold Atmospheric Plasma Jet as a Possible Adjuvant Therapy for Periodontal Disease

**DOI:** 10.3390/molecules26185590

**Published:** 2021-09-15

**Authors:** Gabriela de Morais Gouvêa Lima, Aline Chiodi Borges, Thalita Mayumi Castaldelli Nishime, Gabriela de Fatima Santana-Melo, Konstantin Georgiev Kostov, Marcia Pinto Alves Mayer, Cristiane Yumi Koga-Ito

**Affiliations:** 1 Oral Biopathology Graduate Program, Institute of Science and Technology, São Paulo State University (UNESP), São José dos Campos 12245-000, Brazil; gabrielademorais@yahoo.com.br (G.d.M.G.L.); aline_chiodi@hotmail.com (A.C.B.); gabrieladsantana@yahoo.com.br (G.d.F.S.-M.); 2 Leibniz Institute for Plasma Science and Technology, Felix-Hausdorff Sts. 2, 17489 Greifswald, Germany; thalita.nishime@inp-greifswald.de; 3 Department of Physics, Faculty of Engineering in Guaratinguetá, São Paulo State University (UNESP), Guaratinguetá 12516-410, Brazil; konstantin.kostov@unesp.br; 4 Department of Microbiology, Institute of Biomedical Sciences, Universidade de São Paulo (USP), São Paulo 12247-016, Brazil; mpamayer@icb.usp.br; 5 Department of Environment Engineering, São José dos Campos Institute of Science & Technology, São Paulo State University (UNESP), São Paulo 12247-016, Brazil

**Keywords:** cold plasma, disinfection, periodontitis, *Porphyromonas gingivalis*, biocompatibility

## Abstract

Due to the limitations of traditional periodontal therapies, and reported cold atmospheric plasma anti-inflammatory/antimicrobial activities, plasma could be an adjuvant therapy to periodontitis. *Porphyromonas gingivalis* was grown in blood agar. Standardized suspensions were plated on blood agar and plasma-treated for planktonic growth. For biofilm, dual-species *Streptococcus gordonii* + *P. gingivalis* biofilm grew for 48 h and then was plasma-treated. XTT assay and CFU counting were performed. Cytotoxicity was accessed immediately or after 24 h. Plasma was applied for 1, 3, 5 or 7 min. In vivo: Thirty C57BI/6 mice were subject to experimental periodontitis for 11 days. Immediately after ligature removal, animals were plasma-treated for 5 min once—Group P1 (*n* = 10); twice (Day 11 and 13)—Group P2 (*n* = 10); or not treated—Group S (*n* = 10). Mice were euthanized on day 15. Histological and microtomography analyses were performed. Significance level was 5%. Halo diameter increased proportionally to time of exposure contrary to CFU/mL counting. Mean/SD of fibroblasts viability did not vary among the groups. Plasma was able to inhibit *P. gingivalis* in planktonic culture and biofilm in a cell-safe manner. Moreover, plasma treatment in vivo, for 5 min, tends to improve periodontal tissue recovery, proportionally to the number of plasma applications.

## 1. Introduction

Periodontal disease (PD) is a multifactorial infectious-inflammatory condition that involves a wide range of microorganisms colonizing periodontium. It is influenced by host conditions and external factors that, together, can lead to a shift in the microbial population starting a local inflammatory reaction that activates the immunologic system, proteases, and osteoclasts, causing tissue destruction [1,2].

Around the world, periodontal disease affects 20–50% of the population, being the major cause of tooth loss. Severe PD (4 mm + pocket depth) is more prevalent in older people and in high-income countries due to an increase in life expectation and maintenance of natural teeth [3].

Some bacteria are commonly associated with PD as *Porphyromonas gingivalis*, *Tannerella forsythia*, and *Treponema denticola*. *P. gingivalis*’ ability to influence host immune response is well known and occurs via Toll-like receptors (TLR) (especially TLR-2 and TLR-4) and complement system (triggering Th1 and Th17 cell differentiation) producing interleukin (IL)-17 that is highly involved in osteoclast differentiation and PD progression [4,5]. Recently, it was reported that TLR-9 seems to interact and amplify TLR-2 signaling, contributing to *P. gingivalis* induced periodontal destruction [6].

Periodontal therapies are well established and have demonstrated good results, though still there are limitations regarding the traditional root planing and scaling such as short-term recolonization and no lost tissue gain, even when surgical treatment is applied [7]. Moreover, antibiotic therapy, commonly prescribed during the treatment, has undesirable side effects. Literature shows an interesting secondary periodontal effect on different drugs for systemic heath issues, and molecules that could modulate host response, but none of them demonstrated potential of clinical administration [8,9,10]. Antioxidant therapies have been studied with good results as well, but still transition to clinical application seems to be distant [11]. 

Over the last decade, the application of cold atmospheric plasma (CAP) has become a reality in different fields, like food decontamination, surface modification, wound healing, among others has become a reality. Thus, the literature review led us to believe that the antimicrobial, anti-inflammatory and wound healing stimulation characteristics of cold plasma make it a potential adjuvant therapy in periodontal disease.

Plasma, usually referred to as the fourth state of matter, is a complex mixture of electrons, ions, molecules and atoms in different states, that interact with electrical and magnetic fields and is also an efficient source of highly reactive species [12]. Cold atmospheric plasmas (CAPs) have been extensively studied due to their ability to operate at or very close to room temperature or very close to this and for being biologically safe [12]. Synergetic action of moderate heat, UV radiation and reactive species (e.g., oxygen: ROS, nitrogen: RNS) has been commonly identified as responsible for the plasma antimicrobial activity and the influence of each of these different factors can vary according to the device parameters [4,13]. Plasmas operating in atmospheric air are very efficient in producing hydroxyl and oxygen groups that are frequently appointed indicated as the most important germ inactivation agents leading to the plasma efficiency and devices operating in atmospheric air are very efficient in producing them [14]. In recent years, CAP treatment has been successfully applied in the biomedical field for antimicrobial, healing, and even antineoplastic purposes [5,15,16,17].

Studies on the application of CAP in the treatment of periodontal disease are scarce. Mahasneh et al. [18] and Liu et al. [19] found an inhibitory effect of CAP on *P. gingivalis* planktonic and biofilm cultures, respectively. Xiong et al. [20] showed that *P. gingivalis* biofilm inhibition by CAP (He/O_2_) occurs in the whole extension of a biofilm with 15 μm of thickness. Kleineidam et al. [21] and Eggers et al. [17] demonstrated that CAP, operated with atmospheric air, can improve cell migration and proliferation, upregulate inflammatory cytokines involved in wound repair, and accelerate wound closure after a 60-s in vitro plasma application, in vitro. These studies were conducted in ligament periodontal cells and osteoblast-like cells.

Arndt et al. [22,23] observed increased production of antimicrobial peptides from β-defensins family by keratinocytes and fibroblasts after 2-min exposure to microwave plasma torch (MicroPlaSter ß plasma torch system). Additionally, increased induction of Type I collagen and MCP-1 were detected with no evidence of apoptosis induction. Brun et al. [24] observed increased fibroblast migration and proliferation induced by ROS and consequent (PPAR)-γ expression. No differences in interleukin (IL)-1β, TNF-α or TGF-β levels were noted. 

In vivo experiments are an essential step for standardization of the physical parameters (e.g., plasma temperature, generated UV radiation and oxidative species, and electromagnetic compatibility) for biomedical applications, and most importantly, to elucidate cell and tissue response to plasma generated species, intracellular interactions and possible DNA damage [4]. 

The complexity of PD pathogenesis is a great challenge for clinical treatment success and the synergy among antimicrobial, anti-inflammatory and tissue reparative effects of CAP has motivated us to study this promising therapy on PD in vitro and in vivo, elucidating its effect on periodontal tissue, cells and pathogens.

## 2. Results

### 2.1. Screening of CAP Effect on P. gingivalis by Inhibition Halo Methodology

**Figure 1:**
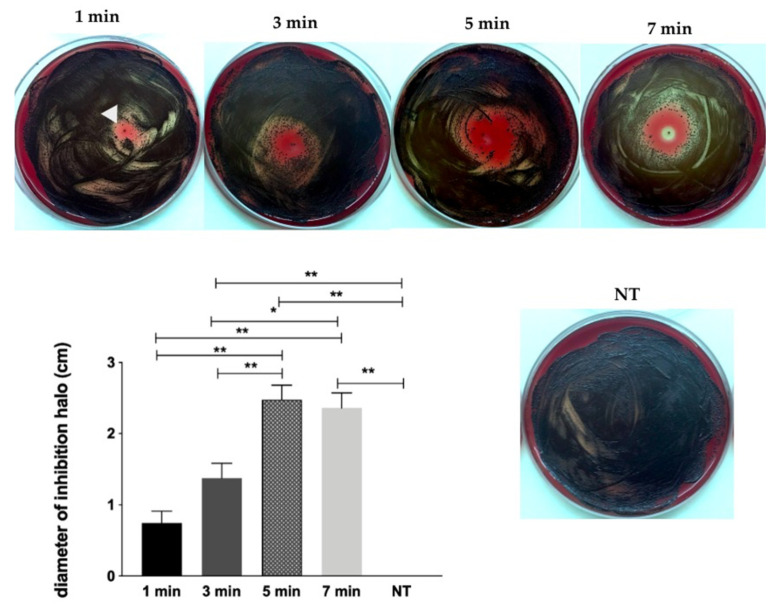
Visual difference of the inhibition halo produced by cold atmospheric plasma (CAP) exposure (arrowhead) of *Porphyromonas gingivalis* culture in all tested periods. Graph of Mean and standard deviation (SD) of the inhibition halos observed after exposure to CAP for 1, 3, 5, and 7 min; (*) *p* < 0.05; (**) *p* < 0.01; NT = non-exposed group.

Figure 1 illustrates the results of bacterial growth inhibition (halo) tests. CAP was able to inhibit *P. gingivalis* growth in a time dependent manner. There was significant difference among the groups, except between 1 and 3 min, and 5 and 7 min groups. 

### 2.2. Effect of CAP on P. gingivalis and Streptococcus gordonii Dual Species Biofilms

#### Colony Forming Units (CFU) Counting

**Figure 2:**
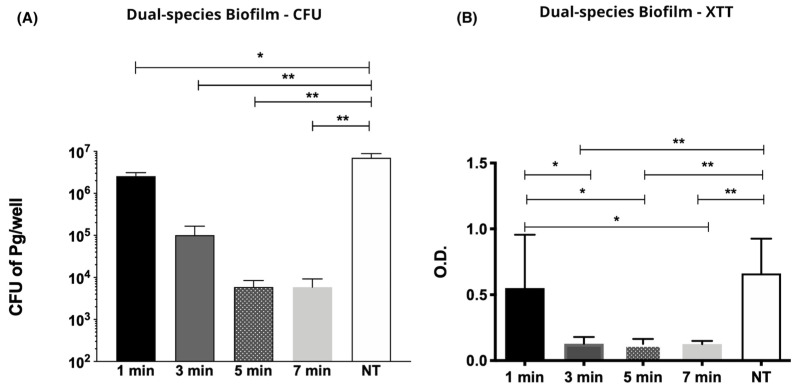
(**A**) Graph of mean and standard deviation values of *Porphyromonas gingivalis* colony forming units per biofilm (CFU/biofilm) after exposure to CAP for 1, 3, 5 and 7 min. (**B**) Graph of mean and standard deviation values of *Porphyromonas gingivalis* OD after XTT reaction according to exposure periods: 1, 3, 5 and 7 min. (*) *p* < 0.05; (**) *p* < 0.01; NT= non-exposed group.

CAP inhibited *P. gingivalis* and *S. gordonii* dual species biofilm in time dependent manner. There was significant reduction in *P. gingivalis* counts (CFU/well) after CAP 3-min exposure (Mean/SD) (1.01 × 10^5^/1.90 × 10^5^) compared to the unexposed group (*p* < 0.05). Mean (SD) of 5.92 × 10^3^ (7.5 × 10^3^) CFU and 5.89 × 10^3^ (10.1 × 10^3^) CFU were achieved for the groups exposed to CAP for 5 min and 7 min, respectively, as shown on Figure 2.

Evaluation of biofilm metabolic activity, assessed by optical density (OD), from biofilms treated for 1, 3, 5, and 7 min after calorimetric XTT assay is presented in Figure 2. When compared to the untreated specimen OD (Mean/SD) (0.662/0.265) there was a significant reduction on cell viability starting from the 3-min application group with OD reading (Mean/SD) of (0.128/0.052) followed by (0.127/0.038) for 5 min, and (0.126/0.023) for 7 min.

### 2.3. Cytotoxicity Evaluation

**Figure 3:**
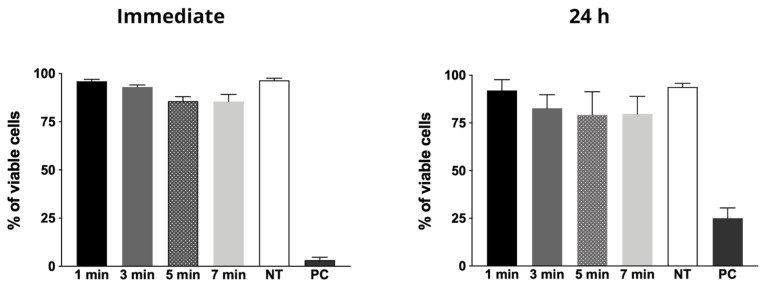
Graphs of mean and standard deviation of cell viability percentages immediately and 24 h after exposure to cold atmospheric plasma. NT = non-exposed group; PC = Positive control (cells exposed to CAP under known cytotoxic parameters for 1 min).

For the tested experimental parameters (e.g., plasma jet power, voltage signal and frequency and amplitude, duty cycle, gas flow rate, and plasma plume distance to the treated surface) the CAP generated with Helium, was not cytotoxic in all tested time periods, both immediately after exposure and 24 h after treatment. For all time periods, except for positive control, cellular viability was about 90%, as shown on Figure 3. 

### 2.4. In Vivo Experiments

#### 2.4.1. Histomorphometry

**Figure 4:**
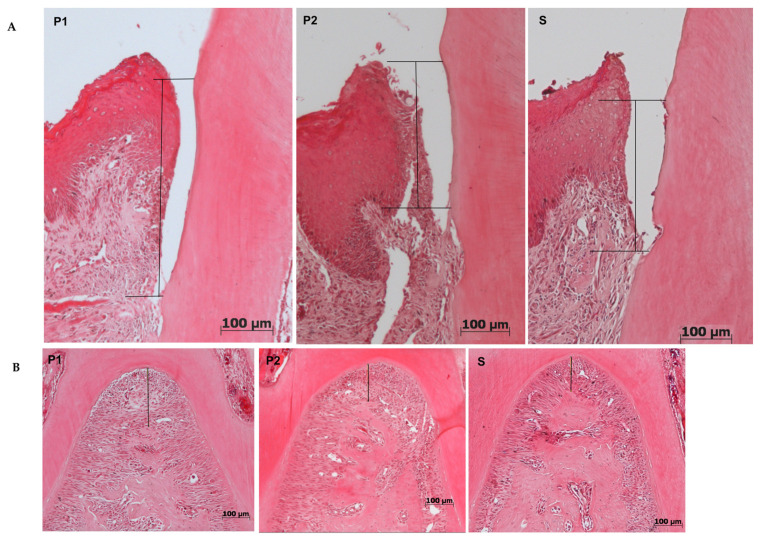
Illustration of the attachment loss: (**A**) ECJ-JE distance; (**B**) Furcation attachment loss in HE-staining among the groups. S—Scaling and root plaining group; P1—Plasma treatment once; P2—Plasma treatment twice.

For both linear periodontium loss analyses, furcation, and attachment loss (CEJ-JE) and for collagen percentage, there were no significant differences among the groups (*p* = 0.96), as shown on Figure 4, despite there was a tendency to attachment loss and collagen recovery for P2 group, as shown on Table 1.

#### 2.4.2. Microcomputed Tomography (Micro-CT)

**Figure 5:**
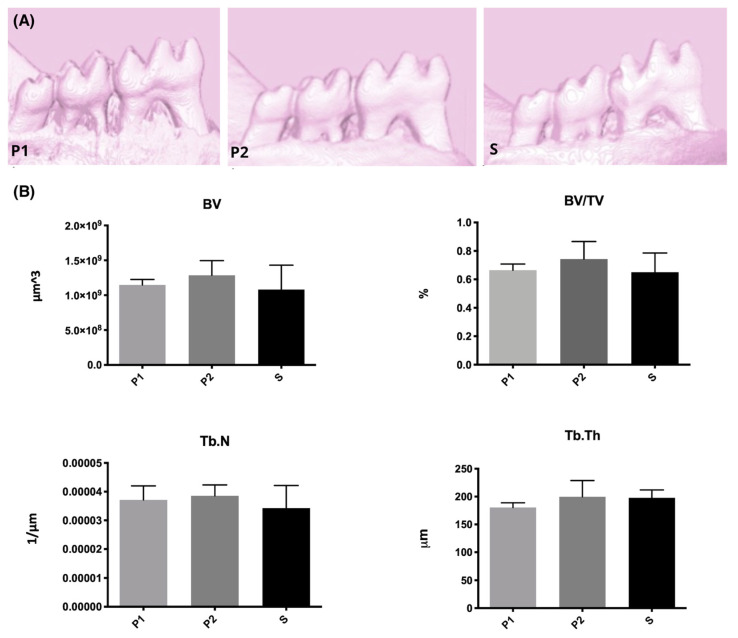
(**A**) 3D reconstruction of the studied groups; (**B**) Graphs of mean and SD for bone volume (BV), bone volume fraction (BV/TV), Trabecular thickness (Tb.Th) and Trabecular number (Tb.N). S—Scaling and root plaining group; P1—Plasma treatment once; P2—Plasma treatment twice.

For all parameters analyzed by micro-CT scanning, there was no difference among the groups, despite Group P2 presented better bone architecture according to the results, with a tendency to periodontal tissue recovery. Figure 5 illustrates 3D reconstruction of the studied groups and graphs of mean and SD for bone volume (BV), none volume fraction (BV/TV), Trabecular thickness (Tb.Th) and Trabecular number (Tb.N). 

For all analysis, histological and micro-tomographic, groups were first compared to a non-disease/not-treated group to validate the experimental protocol. All experimental groups differed significantly from this control group (data shown in Figure S1) proving successful induction of experimental periodontitis.

#### 2.4.3. Histological Effects of Plasma on Health Tissues

Periodontal structures (cementum, alveolar bone, and periodontal ligament) did not suffer any histological alteration when exposed to CAP under the parameters adopted for in vivo tests. There was no sign of inflammation in the furcation area, and the alveolar bone was intact in all its entire extension. There was no abnormal osteoclast activity or morphologic alterations on osteoblast and osteocytes. Cementum layer was intact as well. These results suggest that under the tested conditions, 5 min of CAP exposure does not lead to any histologic alterations 24 h after application. There was no histological difference between treated and untreated health tissues.

## 3. Discussion

As an inflammatory disease, periodontitis has a background of oxidative stress that, according to host response, leads to tissue destruction or no tissue destruction. Recent studies have demonstrated that hyperactivated neutrophils may be involved in individual tendency to develop periodontal disease by exacerbating oxidative stress [11]. 

As oxygen species can influence on microorganism inactivation and at the same time be harmful to host cells, their precise balance in periodontal environment may help on periodontitis treatment. Therefore, the main goal to be achieved by a periodontal therapy is to successfully resolve inflammation pathways modulating phenotype and personalizing treatment [10].

This is where plasma treatment could work as an adjuvant therapy in periodontal disease, assisting in the redox balance and consequent inflammation control. Rezaeinezhad et al. [25] observed that plasma application for 600 s could decrease oxidative biomarkers and pro-inflammatory cytokines in diabetes model, in vitro and in vivo. Periodontitis is highly associated with *diabetes mellitus* and successful periodontal treatment seems to improve glycemic levels. As non-surgical periodontal therapy (e.g., root planing and scaling) has its limitations, adjuvant therapies are welcome and have been widely studied. 

The traditional therapies have limitations as early recolonization, poor attachment and tissue gain, as well as common necessary prescription of antimicrobial drugs. Cold atmospheric plasma has been an efficient inactivation agent against red complex bacteria, especially against *P. gingivalis* [18,26,27,28], and the same results could be expected in our study. Our results show that He-CAP jet was effective against both planktonic growth and dual-species biofilm (*P. gingivalis + S. gordonii*) from 1 to 7 min-application in a distance of 1.5 cm. 

*P. gingivalis* was the bacteria chosen for our study, because of its capacity to invade and influence host immunity leading to PD progression [2,29,30]. Our choice for a duo-species biofilm, on the other hand, was based in preliminary experiments (data not shown), where dual-species biofilms were thicker and allowed higher cell recovery of *P. gingivalis* ATCC 33277 strain.

In addition to the antimicrobial results, CAP has demonstrated biostimulator effects that are desirable in periodontitis therapy as the conventional treatment does not recover the lost periodontium integrity. Other therapies, such as drugs that act on bone metabolism [8,9,31] and antioxidant formulations [11,32], have been suggested for PD treatment with good results. Proresolving lipid mediators reversed microbial dysbiosis and induced tissue healing [10]. Despite the promising results, these new alternatives have not been established as clinical therapy so far. 

Brun et al. [24] studied hematopoietic stem cells and observed plasma-induced proliferation and migration through intracellular ROS formation with no increase on proinflammatory cytokines while Arndt et al. [22] reported an increase in Type I collagen induction for 2 min application of microwave argon plasma torch (MicroPlaSter ß plasma torch system) with argon. Though our in vivo experiments did not show a significant difference in tissue gain, a clear tendency of mineral tissue improvement by the two consecutive plasma treatments, twice for 5 min, can be seen in all graphs related to 3D analysis (micro-CT). Additionally, a slight (non-significative) difference in Type I collagen, evidenced by picrosirius staining can be observed in Figure 4. 

No cytotoxic effects, as well as no physiological and histological alterations induced by in vivo treatments, were observed. Cellular effects and bacterial tolerance can vary accordingly to the plasma source [33]. The device used in this study has demonstrated promising results in microorganism inactivation followed by no or minimum cytotoxicity [26,34], which are in accordance with other studies. Histologically, concordant results were demonstrated by Liu et al. [26], which applied CAP generated with He/O_2_ in oral mucosa of health rabbit for 10 min with no visible alterations.

To the best of our knowledge, the present study is the first one to evaluate CAP in an experimental model of periodontitis in mice and the C57bl/6 mice were chosen due to our previous experience with rodents and because of C57bl/6 being an inbred strain of lab mouse commonly used in PD studies. Zhang et al. [28] performed a study in rats where periodontitis was established for 4 weeks, then plasma treatment was performed in association with root scaling and planing, and euthanasia occurred 7 or 30 days after treatment. In 7 days, plasma treatment for 2 min showed a tendency to improve periodontal tissue loss with no statistical significance, as also observed in our study. Authors could prove the improvement only 30 days after treatment.

Two recent studies in the literature involving CAP are one on *P. gingivalis* and the other, an in vivo study on CAP in tissue destruction and red-complex bacteria. Both focused on periimplantitis [27,35,36]. Though periodontitis and peri-implantitis are similar, with an inflammatory and tissue destructive background, they have importantly different histological and host response features, especially in experimental studies [37,38].

Shi et al. [35] evaluated the effects of CAP exposure (3 min) as an adjuvant to clinical treatment of ligature-induced peri-implantitis in beagle dogs. Clinical and bone analysis (micro-CT and histology) showed better recovery in CAP group 3 months after treatment. *P. gingivalis* and *T. forsythia* counts were significantly reduced when compared to non-exposed control and baseline. A significant decrease in *A. actinomycetemcomitans* counts was detected in the first follow up after one month, but this reduction was not maintained until the second follow up (2 months). Considering the results, the literature and the different aspect of periodontitis and peri-implantitis, mainly the aggressiveness of the last one in experimental models, this finding led us to consider that a longer period between the end of plasma treatment and euthanasia, could bring more expressive and significant differences among our study groups.

Based on the reported favorable results, we can conclude that CAP can be used as adjuvant therapy for periodontal disease, reducing the use of antimicrobial anti-inflammatory drugs, and their consequent adverse effects, lowering local and systemic inflammation and possibly improving mineral tissue impact.

## 4. Materials and Methods

### 4.1. Plasma Jet 

**Figure 6:**
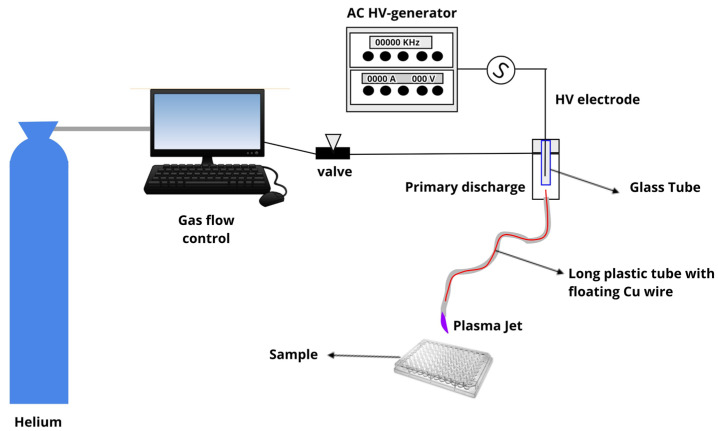
Scheme of the experimental setup (plasma module) used in this study.

The experimental setup used in this study was described previously [39,40,41,42]. The device consists of a 2.3-mm-diameter copper rod connected to a Minipuls4 AC power supply (GBS Elektronik GmbH, Radeberg, Germany). The rod was inserted into a semi-closed quartz tube that was placed on the axis of a syringe-like enclosure made of Delrin, thus forming the primary dielectric barrier discharge (DBD) reactor. A 1.0-m-long flexible polyurethane tube (standard nasogastric feeding tube) was connected to the Delrin enclosure exit nozzle. Inside this tube, there is a floating copper wire in a way that penetrates a few millimeters inside the primary reactor and terminates shortly before the tube tip. Helium (He) with 99.5% purity at a flow rate of 1.0 standard liter per minute (slm) was fed in the primary reactor and expelled in the air through the nasogastric tube end. DBD discharge is generated inside the primary reactor (around the encapsulated high voltage electrode) and due to the potential acquired by the floating electrode, a small (cm-long) plasma jet is ignited at the tube exit and launched into the surrounding air. This remote plasma plume produced at the tip of the long plastic tube can be held by hand and easily directed to a target thus facilitating jet manipulation. In the present study, the plasma jet was powered by a voltage signal with a frequency of 31 kHz and amplitude of 10 kV using burst mode (voltage amplitude modulation) with a duty cycle of 22%. The plasma plume was placed at a 15 mm distance to the treated surface and the mean discharge power calculated at these conditions was 0.6 W. Figure 6 shows a schematic illustration of the plasma jet module used in this study.

### 4.2. Screening of CAP Effect on P. gingivalis by Inhibition Halo Methodology

For screening the effect of CAP on *P. gingivalis* (ATCC 33277), bacteria were cultured in brain heart infusion (BHI) agar supplemented with hemin (5 μg/mL) and menadione (1 μg/mL) for 7 days at 37 °C, under anaerobic conditions. A bacterial standardized suspension containing 1 × 10^8^ CFU/mL was prepared spectrometrically (λ = 600, DO = 0.1 ± 0.05). An aliquot of 100 μL of the suspension was seeded on Fastidious Anaerobe Agar (FAA) plate supplemented with 5% sheep blood, hemin and menadione. CAP was applied at normal incidence, directly on the plate for different times of exposure: 1, 3, 5, and 7 min. Plates were incubated in an anaerobiosis chamber with BD GasPak^™^ gas generator sachet (New Jersey, USA), at 37 °C for 7 days, when growth inhibition halos were measured. The experiment was performed in triplicate, three independent times (*n* = 9). 

### 4.3. Effect of CAP on Dual Species Biofilm of P. gingivalis and Streptococcus gordonii

#### 4.3.1. Colony Forming Units (CFU) Counting

*S. gordonii* (ATCC 10558) was grown in BHI agar, in a 5% CO_2_ chamber at 37 °C for 48 h. Standardized suspension (λ = 600, DO = 0.520 ± 0.02) was obtained in BHI broth supplemented with hemin (5 μg/mL) and menadione (1 μg/mL). Then, 2 × 10^7^ CFU/mL were added to each well of a 96-well plate that was incubated for 24 h at 37 °C in a microaerophilic environment (5% CO_2_). After this, a suspension of *P. gingivalis* (ATCC 33277) (λ = 560, DO = 0.5 ± 0.02) was obtained in spectrophotometer. Pre-established *S. gordonii* biofilm was then washed twice with sterile saline solution (0.9% NaCl) and ~2 × 10^7^ CFU/mL of *P. gingivalis* was added to each well. Biofilms were kept in an anaerobiosis jar with BD GasPak^™^ gas generator sachet, for 48 h at 37 °C. After this period, the biofilms were washed twice with sterile physiologic solution, that was aspirated, and biofilms were treated dry with CAP for 1, 3, 5, and 7 min. A non-exposed (negative control) group was included. 

Biofilms were mechanically dispersed, diluted and plated on FAA supplemented with 5% blood, hemin and menadione. After a 7-day incubation in an anaerobiosis jar with BD GasPak^™^ sachet (New Jersey, USA) (37 °C), characteristic colonies of *Porphyromonas gingivalis* were counted and the number of colonies forming units (CFU) per biofilm was calculated. Experiments were performed in triplicate on three different occasions (*n* = 9). 

#### 4.3.2. Evaluation of Dual Species Biofilm Metabolic Activity by XTT Assay

*P. gingivalis* and *S. gordonii* biofilms were grown in 96-well plates, according to the aforementioned methodology. After exposure to CAP for 1, 3, 5, 7 min, XTT solution (1 mg/mL) with menadione (0.7 mg/mL in ETOH) was added in a 96-well plate. Plates were incubated in aluminum foil in a 5% CO_2_ chamber, for 3 h, and then they were read with a spectrophotometer at 494 nm (Bio-Tek, Synergy HT, Vermont, USA). 

### 4.4. Cytotoxicity Evaluation

The cytotoxicity of the CAP to primary human gingival fibroblast cell line (HGF) and Vero cells was evaluated. Cells were cultivated in DMEM with 10% fetal bovine serum (Gibco, Massachusetts, USA) and supplemented with 1% penicillin/streptomycin (Gibco Massachusetts, USA). Keratinocytes (OBA-9) were cultivated in KSF-M medium (Gibco Massachusetts, USA) supplemented with Defined KSFM Growth Supplement, gentamicin (1/1000), penicillin/streptomycin (1/100), and amphotericin B (1/1000). Cells were kept in a humidified chamber with 5% CO_2_ at 37 °C. Trypan Blue staining was chosen to verify cell viability. Approximately 3 × 10^4^ human gingival fibroblasts (HGF) were plated per well in a 96-well plate and incubated for 24 h in a CO_2_ chamber at 37 °C. Afterwards, the cells were washed in Hanks’ Balanced Salt solution (HBSS) and 50 μL of fresh HBSS were added, a sufficient amount to keep cells humidified during treatment. Cells were exposed to CAP for 1, 3, 5, 7 min. Non-exposed cells were kept as a negative control. Vero cells exposed to CAP under known cytotoxic parameters for 1 min, were used as a positive control [41]. Cells were released with trypsin, immediately or 24 h after treatment, stained with Trypan Blue and spread in a glass slide covered by a glass slide. One hundred cells were counted per slide in an optical microscope in 400× magnitude. The percentage of viable cells (not stained) was calculated. All experiments were performed in triplicate and repeated twice (*n* = 6). According to ISO 10993-5, assays with cell viability of over 70% are considered not cytotoxic [43].

### 4.5. In Vivo Experiments

#### 4.5.1. CAP Effects on Experimental Periodontitis Induced in c57bl/6 Mice

This study was approved by the local Committee on the Ethics in Animal Experiments of the Institute of Science and Technology, São Paulo State University under protocol 05/2015-CEUA-ICT-SJC-UNESP, and it agrees with the National Council for animal experiments and international guidelines.

Animals were caged in groups of five, with food and water ad libitum, with a 12-h light/dark cycle in a temperature-controlled environment. All invasive procedures were performed under sedation with ketamine base (100–150 mg/kg) and xylazine hydrochloride (10–15 mg/kg) IM.

Thirty C57bl/6 female mice, weighing approximately 20 g, were submitted to induction of experimental periodontal disease by ligature, using 1 loop of a 4-0 Nylon suture previously kept for 24 h in a suspension containing 10^8^ CFU/mL of *P. gingivalis* (ATCC 33277). Ligatures were placed around the lower first molars. After this period, the animals were divided into 3 groups according to the treatment (*n* = 10):

RP Group (root planning): Ligature was kept for 11 days, and root planning was carried out on day 11;
P1 Group (plasma 1×): Ligature was kept for 11 days. On day 11, root planning was performed and, after the region was exposed to CAP for 5 min;
P2 Group (plasma 2×) Ligature was kept for 11 days, and root planning was performed. The region was exposed to CAP for 5 min after ligature removal and 2 days after (Day 13) the plasma treatment was repeated.

In addition, a control group (*n* = 5), without induction of periodontal disease, was used to analyze possible effects of plasma on health tissues (in maxillae) and to validate the experimental ligature (mandibulae).

All animals were euthanized on day 15. After euthanasia, hemi-mandibles were fixed in 4% paraformaldehyde. Right mandibles were used for micro computerized tomography (CT) analysis and left mandibles were processed for histological analyses. 

#### 4.5.2. Histomorphometry

For histological analysis, 4 μm slices were stained with Haematoxylin and Eosin (HE)—histomorphometry and Picrosirius Red—quantification of collagen fibers. Two random slices in different depths were analyzed, per animal.

Periodontium slides were digitalized in 100× and 200× magnification for linear periodontium loss analyses. Furcation area was analyzed in 100× magnification and mesial of the first molar in 200×. Measurements from furcation highest point to the top of the bone crest and from cementum–enamel junction (CEJ) to insertion of the junctional epithelium (JE) were made. Image J 1.31p (ImageJ 1.31p; National Institutes of Health, Bethesda, MD, USA) was used to perform the analysis. Mean values of measurements from each sample were statistically compared among the groups.

#### 4.5.3. Microcomputed Tomography (Micro-CT)

Right hemi-mandibles were scanned in a SkyScan 1174 micro-CT scanner (FOP-Unicamp, Piracicaba), with 50 Kvp, 785 μA, 0.7 degrees rotation per image, and 0.5 mm aluminum filter. Images were reconstructed and analyzed by NRecon and CTan software (SkyScan), respectively. A circular ROI was established evolving the edge of the mesial alveolar crest of the first molar and mesial root of the second molar. The volume of 80 slices from furcation roof to apex were analyzed. Bone volume (BV), bone volume fraction (BV/TV), trabeculae number (Tb.N), and trabeculae thickness (Tb.Th) were calculated. 

#### 4.5.4. Histological Effects of CAP on Health Tissues

The control group, without induction of periodontal disease, was used to analyze the possible effects of plasma on healthy tissues. The area around the first molar, on maxillae, were exposed to CAP for 5 min on day 11 and removed after euthanasia. Maxillae were fixed in 4% paraformaldehyde, processed for histological analyses, and 4 μm slices were stained with Haematoxylin and Eosin (HE). 

Fragments were digitalized in 200× magnification and periodontal tissues were analyzed and compared to non-exposed and healthy mandibular fragments. 

### 4.6. Statistics 

Results were analyzed according to data distribution (Shapiro–Wilk test). After, data were compared among the groups using the ANOVA test with Tukey’s post-hoc. Analyses were performed using GraphPad Prism software (GraphPad Software Inc., San Diego, CA, USA). A significance level of 5% was adopted.

## 5. Conclusions

Based on our results, it is possible to conclude that:

- He-CAP jet was effective against planktonic growth and, most important, against dual-species biofilm (*P. gingivalis + S. gordonii*) when applied within 1 to 7 min time interval at 1.5 cm.
- The twice repeated 5 min He plasma jet treatment revealed a tendency of mineral tissue improvement (micro-CT).
- A slight, not significant, amelioration in Type I collagen percentage can be noted after two intercalated 5-min plasma applications.
- CAP generated with Helium had no cytotoxic effects, in all tested time periods, using the adopted experimental parameters (e.g., plasma jet power, voltage signal and frequency, amplitude, duty cycle, gas flow, and plasma plume distance to the treated surface), both immediately after exposure and 24 h after treatment.
- No physiological and histological alterations were induced by the in vivo treatments.

## Figures and Tables

**Table 1:** Mean and standard deviation (SD) of histological periodontal measures; furcation (HE-staining), attachment loss (JEC-JE distance) (HE-staining), and % of collagen (Picrosirius Red-staining).

	P1 (Mean ± SD)	P2 (Mean ± SD)	S (Mean ± SD)
Furcation (μm)	142.0 ± 42.6	147.00 ± 42.3	148.00 ± 44.5
JEC-JE (μm)	242.1 ± 96.9	225.9 ± 61.4	235.6 ± 60.2
% of collagen	12.7 ± 5.9	13.3 ± 5.2	12.9 ± 5.1

S—Scaling and root plaining group; P1—Plasma treatment once; P2—Plasma treatment twice.

## Data Availability

Data sharing is not applicable to this article.

## Supplementary Materials

The following is available online. Figure S1: Histologic and microtomographic measurement data for confirmation of experimental periodontitis induction.

## Sample Availability

Samples of the compounds are not available from authors.
