# Supplementary material for: Cold Atmospheric Plasma Jet as a Possible Adjuvant Therapy for Periodontal Disease
Source: Molecules. 2021 Sep 15;26(18):5590. doi: 10.3390/molecules26185590 (PMC8470429; doi:10.3390/molecules26185590)
Supplement: Supplementary file 1 [file molecules-26-05590-s001.zip › molecules-1354024-supplementary.pdf]

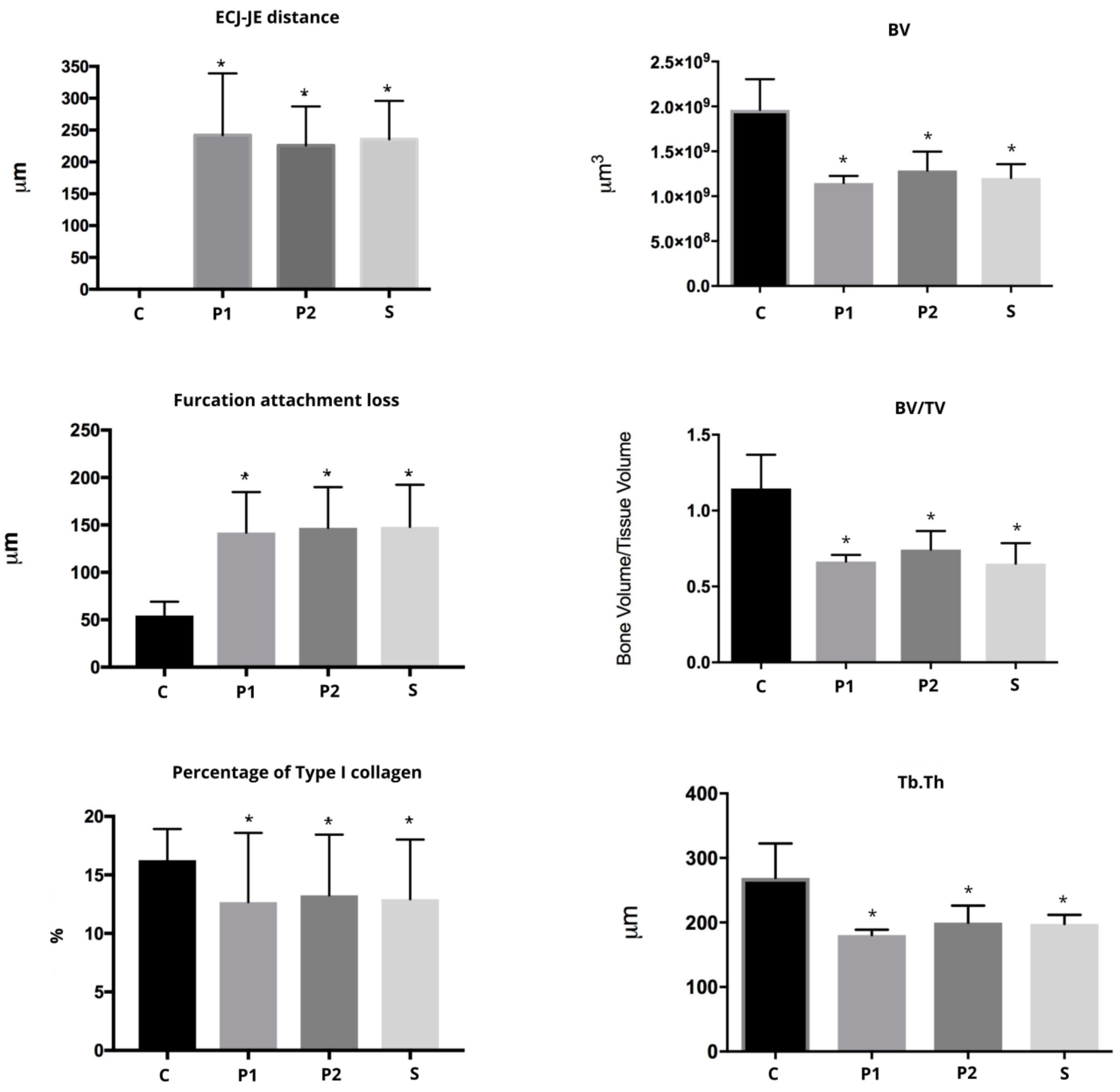

Figure S1. Histologic and microtomographic measurement data for confirmation of experimental periodontitis induction: Graphs of mean and SD for ECJ-JE distance; Furcation attachment loss in HE-staining among the groups, and Percentage of Type I collagen (Picrosirius Red staining); bone volume (BV); bone volume fraction (BV/TV); and Trabecular thickness (Tb.Th). C- Control non-disease/not-treated group; P1- Plasma treatment once; P2 – Plasma treatment twice; S- Scaling and root plaining group. (*) significant difference with Control group.
